# Swimming Suppresses Cognitive Decline of HFD-Induced Obese Mice through Reversing Hippocampal Inflammation, Insulin Resistance, and BDNF Level

**DOI:** 10.3390/nu14122432

**Published:** 2022-06-11

**Authors:** Hu Zhang, Ji-Ling Liang, Qiu-Yue Wu, Jin-Xiu Li, Ya Liu, Liang-Wen Wu, Jie-Lun Huang, Xiao-Wen Wu, Ming-Hui Wang, Ning Chen

**Affiliations:** 1 Graduate School, Wuhan Sports University, Wuhan 430079, China; 17858503393@163.com (H.Z.); ljl19930210@163.com (J.-L.L.); wuqiuyue1252@163.com (Q.-Y.W.); ly13515234752@163.com (Y.L.); hjl19960713@163.com (J.-L.H.); wxw1123wxw@163.com (X.-W.W.); wangminghui290335@163.com (M.-H.W.); 2 Tianjiu Research and Development Center for Exercise Nutrition and Foods, Hubei Key Laboratory of Exercise Training and Monitoring, College of Health Science, Wuhan Sports University, Wuhan 430079, China; ljx973960148@163.com (J.-X.L.); hancock120@163.com (L.-W.W.)

**Keywords:** obesity, swimming, cognitive capacity, inflammation, insulin resistance, BDNF

## Abstract

Obesity is an important public health problem nowadays. Long-term obesity can trigger a series of chronic diseases and impair the learning and memory function of the brain. Current studies show that scientific exercise can effectively improve learning and memory capacity, which also can provide benefits for obese people. However, the underlying mechanisms for the improvement of cognitive capacity under the status of obesity still need to be further explored. In the present study, the obesity-induced cognition-declined model was established using 4-week-old mice continuously fed with a high-fat diet (HFD) for 12 weeks, and then the model mice were subjected to an 8-week swimming intervention and corresponding evaluation of relevant indicators, including cognitive capacity, inflammation, insulin signal pathway, brain-derived neurotrophic factor (BNDF), and apoptosis, for exploring potential regulatory mechanisms. Compared with the mice fed with regular diets, the obese mice revealed the impairment of cognitive capacity; in contrast, swimming intervention ameliorated the decline in cognitive capacity of obese mice by reducing inflammatory factors, inhibiting the JNK/IRS-1/PI3K/Akt signal pathway, and activating the PGC-1α/BDNF signal pathway, thereby suppressing the apoptosis of neurons. Therefore, swimming may be an important interventional strategy to compensate for obesity-induced cognitive impairment.

## 1. Introduction

Obesity, as a sub-health stage with the excessive accumulation of body fat, is mainly affected by genetics and lifestyles. Due to the long-term chronic inflammatory state and abnormal metabolism as the hotbed for metabolic, endocrine, cardiovascular, and nervous system diseases, obesity has become a public health problem that is a current research focus all over the world. Currently, nearly one-third of the world’s population is overweight or obese [1]. According to the Epidemiological Survey in China from 2015–2019, the obesity rate in China reveals a significant increase, and children and adolescents have also become the hardest-hit areas for obesity [2]. Unhealthy diets such as high-fat and high-sugar diets may cause damage of brain tissues, thus resulting in the decline in cognitive and learning capacity [3]. Therefore, reducing high-fat diet (HFD)-induced obesity and alleviating the impaired brain function have become an urgent problem to be solved.

There are many ways to intervene obesity, including exercise, diets, drugs, and surgery. Previous studies have demonstrated that obesity-induced impairment of cognitive and memory function can be suppressed and rescued after exercise [4], ketogenic diet [5], and surgical interventions due to body weight loss [6]. Therefore, the gain of body weight may be the culprit. However, adolescents are in the rapid growth and development stage, and surgical, drug, and dietary interventions may adversely affect the body. Exercise is an economical and green intervention strategy for health promotion, physical fitness, and obesity management. Considering the impact of different exercise methods on obesity, swimming intervention that is more suitable for obesity was selected in this study. In addition, previous studies on molecular mechanisms have shown that the decline in cognitive function may be correlated with insulin resistance, neurotrophic factor deficiency, apoptosis, and reduced synaptic plasticity in obesity models, as well as the long-term chronic inflammation at the status of obesity [7]. Insulin resistance in the hippocampus is an important factor that leads to the decline of cognitive function. Insulin resistance may also indirectly result in the adverse impact on synaptic plasticity by reducing the brain-derived neurotrophic factor (BDNF), eventually leading to the decline of cognitive and memory function [8]. Many studies using HFD-induced obesity mouse models have also documented the increased inflammation, insulin resistance, and reduced BDNF level in the hippocampus [9,10,11,12]. Therefore, reduced inflammation, increased insulin sensitivity, up-regulated BDNF, and suppressed apoptosis of neurons may be the critical molecular mechanisms for ameliorating obesity-induced cognitive decline during the process of body weight loss.

It is well known that scientific and reasonable exercise can significantly promote the health of the body and reduce the occurrence of sports injuries. Relevant studies have shown that swimming can effectively improve learning and memory functions and increase hippocampal BDNF level [13]. Therefore, in this study, the relevant indicators associated with inflammation, insulin resistance, BDNF level, and apoptosis in hippocampal tissues were detected to uncover the molecular mechanisms for alleviating obesity-induced cognitive impairment upon a swimming intervention in HFD-induced obese mice.

## 2. Materials and Methods

### 2.1. Animals, Experimental Design, and Ethics

**Figure 1:**
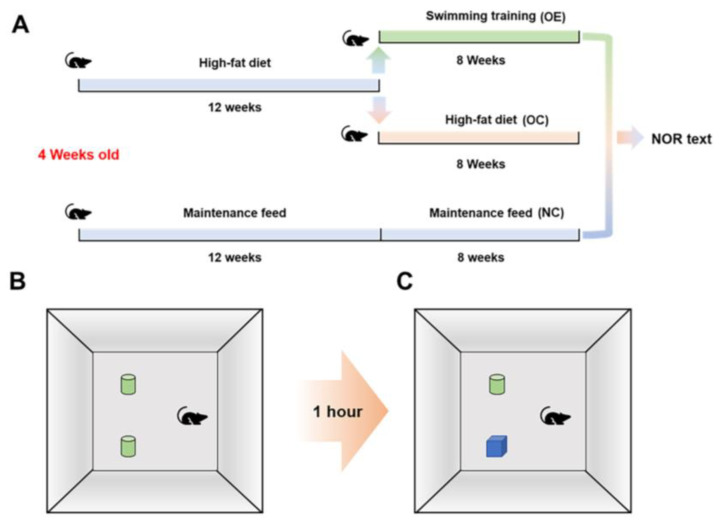
The diagram for describing animal grouping with corresponding feeding and exercise intervention, as well as new object recognition test. (**A**) Flow chart of mouse grouping with corresponding feeding and exercise intervention; (**B**) A 10-min test environment adaptation; (**C**) learning and memory capacity test.

Thirty 4-week-old specific pathogen-free (SPF) grade C57BL/6 male mice (body weight: 19.9 ± 1.4 g) were purchased from the Experimental Animal Center of Hubei Provincial Center for Disease Control and Prevention (Certificate No. 42010200005338). The mice were randomly divided into the regular diet group (10 mice) as the normal control (NC) group and the HFD group (20 mice) to establish the obesity model. The obese mice were then divided into two subgroups, which were the obesity-control (OC) group without swimming and the obesity-exercise (OE) group with loading-free swimming for 8 weeks (Figure 1A). The mice involved in this study were kept under the conditions with a standard light and dark cycle (12:00–12:00) and temperature (22 ± 2 °C). The regular diet had the energy ratio of 63.4% from carbohydrates, 22.8% from protein, and 13.8% from fat, and the high-fat diet had the energy ratio of 60% from fat, 20% from protein, and 20% from carbohydrates.

### 2.2. Swimming Training Protocol

The obese mice were subjected to non-intermittent swimming training in a swimming tank (30 ± 1 °C) without weight bearing for 8 weeks, five times a week and 60 min each time. In order to relieve the stress from swimming, the mice were provided with incremental exercise time for adaptation from 15, 20, 30, 45, and 60 min in the first week. The swimming time was set up in the early morning in accordance with the mouse biological clock (18:00–20:00). During the exercise intervention in this study, all mice were abstained from other additional physical stimulations.

### 2.3. New Object Recognition Test

After the swimming intervention for 8 weeks, all mice were subjected to a new object recognition test for evaluating the learning and memory function. The mice to be tested were placed in the test environment for 24 h in advance for environmental adaptation, and the training began to place two identical objects on the same side. The mouse was placed in the field with its back facing the two same objects, and the heads of the mice were at the same distance from the two objects. After the mice were put into the test environment for 10 min to adapt to the test environment, the mice were immediately put back into the rearing box, and after 1 h of rest, one of the objects was replaced with another object with a different color and shape to conduct new object recognition tests within 5 min, as shown in the general process (Figure 1B,C).

### 2.4. Histological Examination of Hippocampal Tissues

After the novel object recognition test, 3 mice in each group were sequentially perfused with 0.9% saline and 4% paraformaldehyde (pH 7.4) under anesthesia. After perfusion, the mice were sacrificed by the dislocation of cervical vertebrae, and the brain was removed and placed in 4% paraformaldehyde at 4 °C overnight for paraffin embedding and staining. The paraffin block of mouse brain tissue in each group was cut coronally into sections with the thickness of 5 μm using a microtome, and the brain tissue sections were then subjected to de-paraffinization and rehydration, Nissl staining, dehydration sealing, and microscopic examination. HE staining and antigen retrieval were performed after de-paraffinization and rehydration, endogenous peroxidase blocking, primary antibody incubation at 4 °C overnight, secondary antibody incubation at room temperature, color development, hematoxylin staining, dehydration, clearing, mounting, and microscopic evaluation. In immunofluorescence, the primary antibody and corresponding secondary antibody, CY3-TSA, were added for the probing. Nuclei were counterstained with DAPI. The images were acquired by an imaging system (Eclipse-E100, Nikon, Japan) and fluorescence was analyzed using ImageJ software (NIH, Bethesda, MD, USA).

### 2.5. Western Blot

The hippocampal tissues of the mice were collected on a low-temperature plate and placed in liquid nitrogen immediately and then transferred to a −80 °C refrigerator for storage and future use. Hippocampal tissue samples were subjected to pre-cooled cell lysis in the presence of protease inhibitors and to homogenization in ice for 30 min. The supernatant was harvested by centrifuging at 10,000× *g* for 5 min at 4 °C. After protein concentration of the supernatant was detected by the BCA method, the aliquots of the supernatant were mixed with 2× loading buffer and then subjected to a metal-plate bath at 95 °C for 5 min to denature proteins. Approximately 25 μg of total protein in the prepared samples were separated using 10% or 12% sodium dodecyl sulfate-polyacrylamide gel electrophoresis (SDS-PAGE) and then transferred to a polyvinylidene fluoride (PVDF) membrane. The target protein in the membrane was probed by a specific primary antibody against IL-6, TNF-α and BDNF (GeneTex, Irvine, CA, USA), p-IRS-1^ser307^, IRS-1, p-Akt^ser473^, Akt, p-PI3K, PI3K, JNK, p-JNK, PSD95, and β-actin (Cell Signaling Technology, Danvers, MA, USA), as well as the corresponding secondary antibody (Cell Signaling Technology, Danvers, MA, USA). Protein bands were visualized by using an enhanced chemiluminescence (ECL) reagent and imaged using an ultra-sensitive fluorescence/chemiluminescence imaging system ChemiScope6300 (CLiNX Science Instruments, Shanghai, China).

### 2.6. Statistical Analysis

All data were expressed as mean ± standard deviation (M ± SD). Statistical analysis was conducted by GraphPad Prism software (La Jolla, CA, USA). A one-way analysis of variance (ANOVA) was used to analyze statistically significant differences between multiple groups for parametric data. Otherwise, a nonparametric Kruskal-Wallis analysis was performed. The significant difference was considered at *p* < 0.05.

## 3. Results

### 3.1. Swimming Reduced Lee’s Index and Body Weight of HFD-Induced Obese Mice

**Figure 2:**
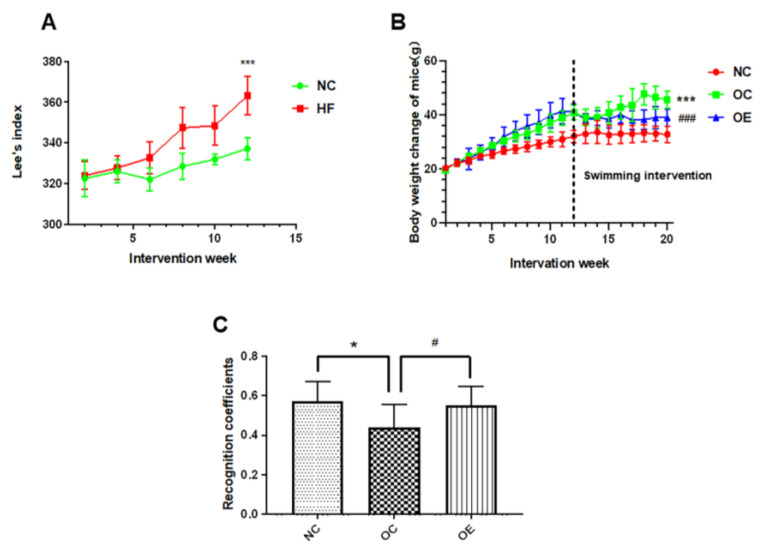
Effect of a swimming intervention on body weights of the mice after 12 weeks of normal chow and high-fat feeding for 8 weeks, as well as the assessment of the learning and memory capacity for the mice through a novel object recognition test. All data were presented as mean ± standard deviation (M ± SD) (*n* = 7 mice per group). (**A**) statistical analysis for the changes in Lee’s index of the mice within the 12-week feeding for establishment of the obesity model; (**B**) statistical analysis of the changes in body weights of the mice in NC, OC, and OE groups upon swimming intervention; (**C**) statistical analysis of recognition coefficients of the mice from the NC, OC, and OE groups, and the recognition coefficient is equal to the times of recognizing new objects to the total times of recognizing old and new objects. Compared with the NC group, * *p* < 0.05, *** *p* < 0.001; compared with the OE group, ^#^ *p* < 0.05, ^###^ *p* < 0.001.

In order to examine the effect of HFD on physical development and body weight, we regularly measured the body weights and the trunk length from the nose to the anus of the mice in each group once every two weeks to calculate Lee’s index (Figure 2A). Compared with the normal control (NC) group, the mice from the HFD group revealed extremely significant differences in Lee’s index (*p* < 0.001) from 6 to 12 weeks and extremely significant differences in body weight from 4 to 20 weeks (*p* < 0.001), indicating that HFD can rapidly promote the body development of the mice. After obesity modeling for 12 weeks, the obese mice were divided into the obesity-exercise (OE) group and the obesity-control (OC) group. Compared with the OC group, the body weights of the mice from the OE group showed a significant decrease after a swimming intervention for 8 weeks (*p* < 0.05) (Figure 2B). Therefore, 12 weeks of HFD administration significantly induced the development of obesity in young mice, while the 8-week swimming intervention reduced the body weights of the obese mice.

### 3.2. Swimming Intervention Enhanced Learning and Memory Capacity of Obese Mice

Usually, learning and memory functions reveal the rapid development during the adolescent period, and the long-term consumption of high-fat diets has adverse effects on the brain and nervous system [14]. Therefore, we used a novel object recognition test to evaluate the changes in learning and memory capacity of obese mice (Figure 2A,B). Compared with the NC group, the recognition capability to novel objects of the mice from the OC and OE groups was significantly lower, indicating that a high-fat diet could suppress the learning and memory capacity of the mice (*p* < 0.05). In contrast, after a swimming intervention for 8 weeks, the learning and memory capacity of the mice from the OE group was significantly higher than that in the OC group (*p* < 0.05) (Figure 2C). These results suggest that the long-term consumption of a high-fat diet during the adolescent period can lead to the impaired learning and memory capacity of the mice, while a swimming intervention can be beneficial to the improvement or rescuing of the declined learning and memory capacity of the obese mice.

### 3.3. Swimming Suppressed Hippocampal Neuronal Degeneration of Obese Mice

**Figure 3:**
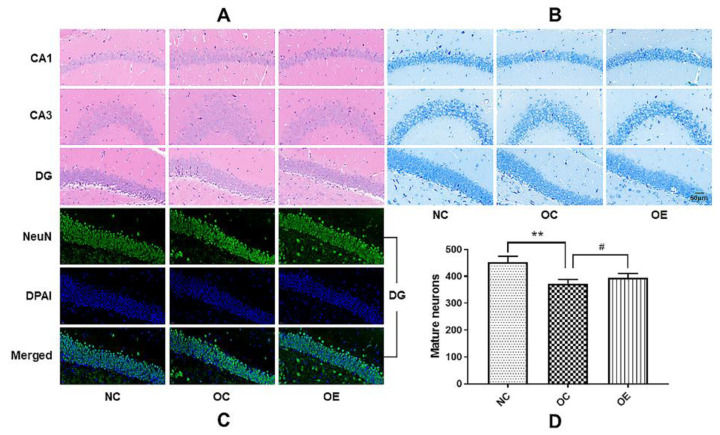
Representative images for the HE, Nissl, and NeuN staining of hippocampal tissues. All data were presented as mean ± standard deviation (M ± SD) (*n* = 3 mice per group). (**A**) HE staining of the CA1, CA3, and DG hippocampal regions of the mice from the NC, OC, and OE groups; (**B**) Nissl staining of the CA1, CA3, and DG hippocampal regions of the mice from the NC, OC, and OE groups; (**C**) NeuN staining of the DG hippocampal region of the mice from the NC, OC, and OE groups. All images were acquired under a 400× optical microscope. (**D**) Statistical analysis of mature neurons in the DG regions of hippocampal tissues from the NC, OC, and OE groups. Compared with the NC group, ** *p* < 0.01; compared with the OE group, ^#^ *p* < 0.05.

Long-term consumption of a high-fat diet may lead to the damage and functional decline of hippocampal neurons [15]. In order to further understand the effect of obesity on hippocampal neurons, we conducted HE, Nissl, and NeuN staining to evaluate the morphological and pathological changes of the hippocampal neurons of the obese mice upon the swimming intervention (Figure 3A–C). Compared with the NC group, the neuronal damage, disordered and sparse neuron arrangement, and decreased number of mature neurons in the mice from the OC group were observed. In contrast, the swimming intervention rescued these obesity-induced impairments or abnormal changes of hippocampal neurons, or it suppressed the hippocampal neuronal degeneration of the obese mice, as shown in the deep and compact neuronal borders and nuclei with Nissl staining (Figure 3B) and more mature neurons under the same background intensity (Figure 3D). These results suggest that obesity can impair the maturation process and the corresponding functions of neurons, while a swimming intervention can ameliorate hippocampal neuronal damage and stimulate the maturation of neurons.

### 3.4. Swimming Suppressed Hippocampal Neuroinflammation of Obese Mice

**Figure 4:**
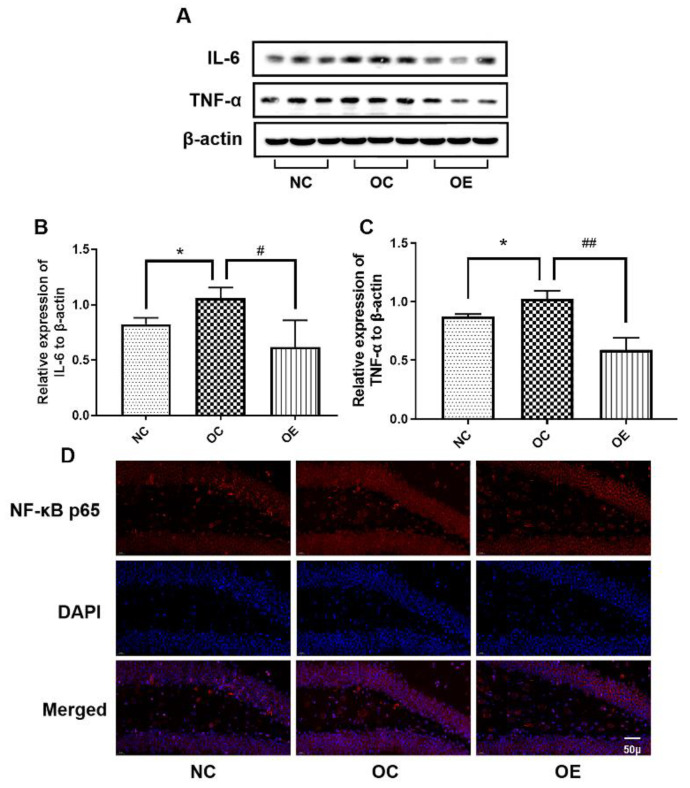
The Western blot of proteins associated with neuroinflammation (**A**) and corresponding statistical analysis of IL-6 (**B**) and TNF-α (**C**) expression levels, as well as the immunofluorescence of NF-κB p65 (**D**) in hippocampal tissues. All data were presented as mean ± standard deviation (M ± SD) (*n* = 3 mice per group). Immunofluorescence images were acquired under a 400× optical microscope. Compared with the NC group, * *p* < 0.05; compared with the OE group, ^#^ *p* < 0.05, ^##^ *p* < 0.01.

Obesity can trigger chronic inflammation, and same changes can be observed in the nervous system [16]. In order to determine the level of inflammation in hippocampal tissue under the status of obesity, we evaluated inflammation-related proteins by Western blot (Figure 4A). Compared with the NC group, the expression of IL-6 and TNF-α in hippocampal tissues of the mice from the OC group showed a significant increase (*p* < 0.05); on the contrary, swimming intervention reversed the obesity-induced increase of IL-6 and TNF-α (*p* < 0.05; *p* < 0.01) (Figure 4B,C). The immunofluorescence results showed that the obviously higher expression of NF-κB p65 in the hippocampal tissues of mice from the OC and OE groups was observed when compared with the NC group; in contrast, swimming intervention down-regulated the expression of NF-κB p65 in hippocampal tissues in comparison with the OC group (Figure 4D), indicating that the swimming intervention has an obviously inhibitory effect on neuroinflammation, thereby executing the suppression of the neuroinflammation-induced reduction of learning and memory capacity.

### 3.5. Swimming Activated Hippocampal Insulin Signaling in Obese Mice

**Figure 5:**
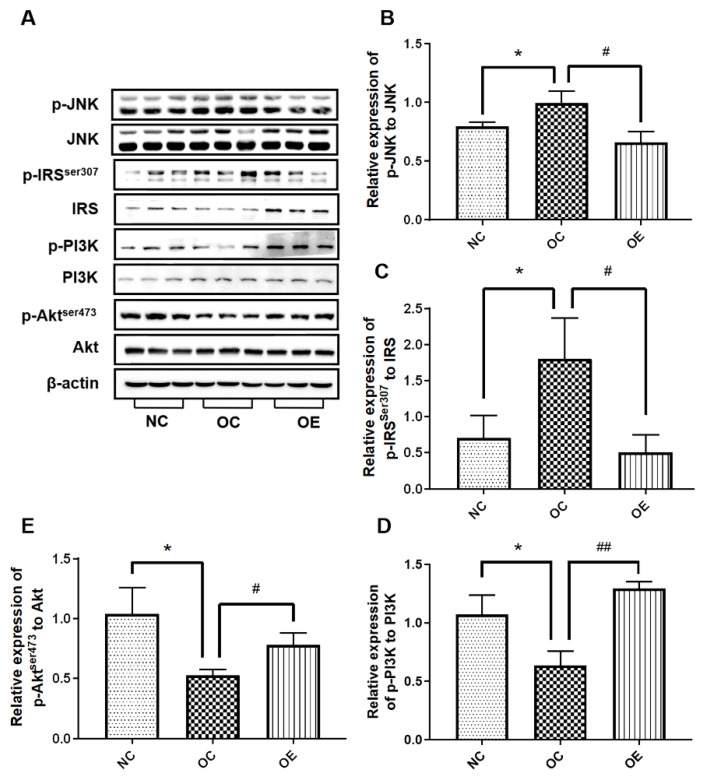
The Western blot of proteins associated with insulin signal pathway (**A**) and corresponding statistical analysis of p-JNK/JNK (**B**), p-IRS-1^ser307^/IRS-1 (**C**), p-PI3K p85/PI3K (**D**), and p-Akt^ser473^/Akt (**E**) ratios in the hippocampal tissues. All data were presented as mean ± standard deviation (M ± SD) (*n* = 3 mice per group). Compared with the NC group, * *p* < 0.05; compared with the OE group, ^#^ *p* < 0.05, ^##^ *p* < 0.01.

Long-term obesity may lead to inflammation in the body, thus inducing insulin resistance and impairing learning and memory functions [17]. To further explore the changes in inflammation and insulin-related signaling in hippocampal tissues of obese mice, Western blotting was used to evaluate the expression of the proteins associated with inflammation and the insulin signal pathway (Figure 5A). Compared with the NC group, p-JNK/JNK and p-IRS-1^ser307^/IRS-1 ratios in hippocampal tissues of the mice from the OC group revealed a significant increase (*p* < 0.05); in contrast, p-PI3K/PI3K and p-Akt^ser473^/Akt ratios exhibited an obvious decrease (*p* < 0.05), suggesting that a high-fat diet contributes to inflammation-induced insulin resistance. On the other hand, the swimming intervention suppressed the increase of p-JNK/JNK and p-IRS-1^ser307^/IRS-1 ratios (*p* < 0.05) and the reduction of p-PI3K/PI3K and p-Akt^ser473^/Akt ratios (*p* < 0.05, *p* < 0.01) (Figure 5B–D), thereby stimulating the activation of the insulin signal pathway. These results suggest that elevated levels of inflammation induced by long-term obesity can lead to insulin resistance in the hippocampal tissue, while a swimming intervention can attenuate inflammation-induced insulin resistance.

### 3.6. Swimming Up-Regulated The Proteins Associated with Neurotrophic Factors and Synaptic Plasticity in the Hippocampal Tissue of Obese Mice

**Figure 6:**
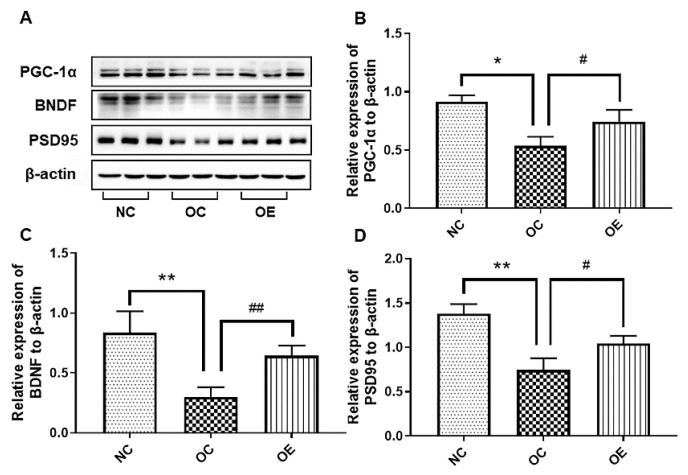
The Western blot of proteins associated with neurotrophic factors and synaptic plasticity (**A**) and corresponding statistical analysis of PGC-1α (**B**), BDNF (**C**), and PSD95 (**D**) expression levels in hippocampal tissues of the mice. All data were presented as mean ± standard deviation (M ± SD) (*n* = 3 mice per group). Compared with the NC group, * *p* < 0.05, ** *p* < 0.01; compared with the OE group, ^#^ *p* < 0.05, ^#^^#^ *p* < 0.01.

The reduction of neurotrophic factors and synaptic plasticity in hippocampal tissues of the mice with obesity may be the inducers for poor learning and memory capacity [18]. To understand the effects of neurotrophic factors on the regeneration of hippocampal neurons in an obese state, we conducted the evaluation of the corresponding protein expression associated with neurotrophic factors and synaptic plasticity in the hippocampal tissue of the mice through Western blot. The expression of PGC-1α, BDNF, and PSD95 in the hippocampal tissues of the mice from the OC group revealed a significant decrease when compared with the NC group (Figure 6A–D) (*p* < 0.05, *p* < 0.01), while a swimming intervention could rescue the down-regulation of these proteins (*p* < 0.05, *p* < 0.01). Therefore, a swimming intervention is beneficial to the rescuing of down-regulated PGC-1α, BDNF, and PSD95 in hippocampal tissues of the mice caused by the long-term consumption of a high-fat diet, thereby enhancing neurotrophic factors and synaptic plasticity.

### 3.7. Swimming Inhibited Hippocampal Neuronal Apoptosis in Obese Mice

**Figure 7:**
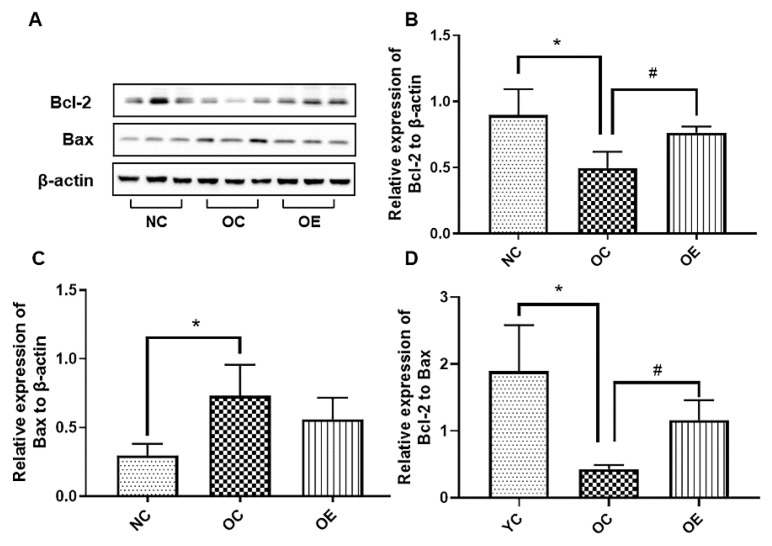
The Western blot of proteins associated with apoptosis (**A**) and corresponding statistical analysis of Bcl-2 (**B**) and Bax (**C**) expression levels, as well as Bcl-2/Bax (**D**) in hippocampal tissues of the mice. All data were presented as mean ± standard deviation (M ± SD) (*n* = 3 mice per group). Compared with the NC group, * *p* < 0.05; compared with the OE group, ^#^ *p* < 0.05.

A high-fat diet can induce the oxidative stress for stimulating apoptosis of the hippocampal neurons in mice, thereby triggering a reduced learning and memory capacity [19]. To further explore the effect of a long-term high-fat diet on apoptosis of the hippocampal neurons in obese mice, we examined the expression of anti-apoptotic and apoptotic proteins. Compared with the NC group, a significant decrease in Bcl-2 and an increase in Bax in hippocampal tissues of the mice from the OC group were observed (*p* < 0.05); on the contrary, a swimming intervention could up-regulate Bcl-2 and down-regulate Bax to execute the suppression of hippocampal neuronal apoptosis (Figure 7A–C). Although Bax did not appear to be significantly different between the OC and OE groups, the expression of Bax in the OE group showed a downward trend when compared to the OC group, and it could significantly reverse the Bcl-2/Bax ratio to suppress apoptosis of the hippocampal neurons in obese mice (Figure 7D) (*p* < 0.05). These results suggest that the long-term consumption of a high-fat diet may trigger apoptosis of the hippocampal neurons, and swimming can rescue this phenomenon to some extent.

## 4. Discussion

Obesity can lead to the impairment of learning and memory capacity, which is confirmed by increasing studies. However, the molecular mechanisms for the impairment of learning and memory capacity caused by obesity are unclear. The 1–6-months-old C5BL/6 mice are equivalent to 12–30-year-old humans during the rapid development period to adult period [20]. Therefore, this study highly mimicked the development of the adolescent population with obesity using 4-week-old young male mouse models fed with a high-fat diet to obesity for 3 months, which can avoid the uncertain factors such as the physiological cycle of female mice as soon as possible. Exercise is an important external means to promote the development of brain function accompanied by the suppression of obesity [21]. However, considering the risk of exercise injury caused by larger body weight, this study adopted a swimming intervention for 2 months. The experimental results were consistent with the expectation. Swimming intervention significantly reduced body weight, alleviated neuroinflammation and insulin resistance, and up-regulated neurotrophic factors to achieve the reversal of learning and memory function in obese mice.

The occurrence of obesity may lead to a significant decline in memory, but some studies have found that the decline in cognitive function caused by a high-fat diet may precede the occurrence of obesity [22]. Relevant studies have shown that short-term [23] and long-term exposure [15] to high-fat diets can result in the obesity-induced impairment of learning and memory capacity. Only 4 days of high-fat and high-sugar diets in humans can impair hippocampal-dependent learning and memory function to some extents [24]. The 7–9-year-old adolescents with the long-term intake of saturated fatty acids present a negative correlation with learning and memory function [25], as confirmed by the same performance in Zebrafish [26], which stimulates the interest in exploring the beneficial effect of regular exercise intervention on the mitigation of the HFD-induced impairment of learning and memory function and precision mechanisms through the mouse model, thereby providing a reference or guidance for health promotion. However, in 12-month-term HFD feeding, rats showed better learning and memory capacity and larger hippocampal volume [27], and the consumption of HFD for 6 months showed no spatial memory impairment [28]. This phenomenon, that a long-term high-fat diet does not trigger the changes in learning and memory capacity, may be related to stress, self-resistance, and the adaptation of neurons to high-fat diets, but the specific mechanisms need to be further explored. Similarly, the level of inflammation from high-fat diets can be altered with a change in the duration of diet interventions [29]. Although there is disagreement about the relationship between the duration of high-fat diet intervention and the impairment of learning and memory capacity, it has been widely reported and recognized that obesity can impair learning and memory capacity.

From the point of view of molecular mechanisms, the impaired learning and memory capacity may be related to the increased level of hippocampal inflammation caused by high-fat diets [8]. It has been widely proven that obesity can increase inflammation in the body, with a similar effect on brain tissue [30]. A cross-sectional study with 10,000 persons in the USA has demonstrated that the level of inflammation is negatively correlated with memory, and it also has the reference value in complex environments [31]. The exposure of juvenile rats to HFD-induced inflammation and reduced learning and cognitive function [32,33]. In a clinical trial with a large volume of samples involving more than 8000 adolescents and children, the results showed that the level of inflammation in obese adolescents is usually accompanied by poor learning and memory capacity [34]. The mice subjected to the consumption of short-term high-fat diets can result in the elevation of pro-inflammatory cytokines such as IL-6 and TNF-α in hippocampal tissues [35]. Once JNK is activated, it can act on NF-κB to enter the nucleus to promote the transcription of inflammatory genes, thus resulting in the release of more inflammatory factors, eventually forming a vicious circle and aggravating the degree of inflammation and impaired learning and cognitive capacity [36]. After reducing inflammation, the dietary intervention can fully or partially rescue the impaired cognition of obese mice. Therefore, reducing inflammation may be a solid inducer to delay or suppress the reduction of learning and memory function [37]. In the present study, the impaired learning and memory capacity of the obese mice was highly modulated by the expression levels of IL-6, TNF-α, NF-κB p65, and JNK in hippocampal tissues, and a regular swimming intervention can significantly down-regulate these inflammatory proteins. This is consistent with the significant reduction of inflammation levels in the hippocampal tissues of animal models subjected to treadmill running, swimming, or voluntary wheeling running interventions [38,39,40].

Inflammation caused by obesity may be an important factor associated with insulin resistance [41]. Previous studies have also documented that adolescent mice fed with high-fat diets present an increased level of inflammation in hippocampal tissues, and insulin signaling is also significantly blocked [42,43]. As a bridge between inflammation and insulin, inflammatory factors promote the phosphorylation of IRS-1 at the Ser307 site by activating JNK phosphorylation, thereby hindering insulin signaling and exacerbating insulin resistance. Long-term chronic inflammation can activate JNK and eventually lead to the occurrence of insulin resistance [44]. Insulin resistance in the hippocampal tissue is considered one of the important triggers for the decline in learning and memory function; therefore, activating the insulin signaling pathway can rescue impaired learning and memory capacity [45]. In our study, the similar results with increased expression levels of the proteins associated with the signal pathways of insulin resistance such as JNK/IRS-1/PI3K/Akt in the hippocampal tissues of the obese mice were observed, and the swimming intervention rescued the abnormal expression of these proteins (Figure 5), further suggesting impaired learning and memory capacity due to the insulin resistance from high-fat diets, and alleviated the obesity state and recovered insulin resistance for enhancing learning and memory capacity upon regular exercise intervention.

In addition, neurotrophic factors and synaptic plasticity are closely related to improving learning and memory functions. In the present study, the expression levels of PGC-1α, BDNF, and PSD95 showed a downward trend in the hippocampal tissues of obese mice, as consistency with the literature reports describing the alleviation of learning and memory impairment caused by soybean oil-induced BDNF reduction [39]. As an important neurotrophic factor, BDNF can play a critical role in the release and reception of neurotransmitters in presynaptic and postsynaptic membranes, thus promoting the connection between synapses and even the regeneration of nerves, thereby improving learning and memory functions. As an intracellular protein, PSD95 plays a vital role in neuronal synaptic plasticity and learning and memory functions [46]. A previous study has also found that exercise can inhibit the reduction of BNDF in the hippocampal tissue of an obesity model [47]. The feeding of high-fat diets for 3-month-old mice can trigger the reduction of the PSD95 level [48], and both aerobic exercise and resistance exercise training can up-regulate hippocampal PSD95 expression [49,50]. Therefore, a swimming intervention for enhancing the learning and memory capacity of obese adolescents may partly depend on the activation of the PGC-1α/BDNF signal pathway.

The long-term consumption of high-fat diets may be detrimental to the survival of hippocampal neurons, and corresponding studies have shown that exercise interventions can promote the improvement of hippocampal function by suppressing the apoptosis of hippocampal neurons, thereby enhancing learning and memory capacity [19]. Exercise can inhibit the apoptosis of hippocampal neurons in obese offspring [51], which is similar to our experimental results with the up-regulated anti-apoptotic protein Bcl-2, down-regulated pro-apoptotic protein Bax, and increased Bcl-2/Bax ratio (Figure 6), as well as the reduced expression of the proteins in hippocampal tissues associated with inflammation and insulin resistance upon the swimming intervention (Figure 4 and Figure 5). However, endoplasmic reticulum stress, mitochondrial dysfunction, elevated ROS level caused by high-fat diets, gut microbiome change, and combinatorial stress responses under different dietary conditions may also be important triggers for neuronal apoptosis [33,51,52,53,54,55]. Consistently, aerobic exercise plays a positive role in reducing hippocampal neuronal apoptosis.

As is well known, dietary modification is another important way to mitigate obesity and enhance learning and cognitive capacity. In order to better understand the effect of exercise intervention on regulating the progression of obesity, in our study, the benefits of exercise intervention were significantly highlighted. Swimming intervention can alleviate inflammatory responses, promote insulin sensitivity, up-regulate neurotrophic factors, increase synaptic plasticity, and suppress apoptosis (Figure 4B,C and Figure 5A), as well as rescue insulin resistance signaling (Figure 5B–D), suggesting that better nutritional supplementation combined with regular exercise training during the rapid development stage of the body may have more benefits, including the rescuing of the high-fat diets-induced impairment of learning and memory capacity.

## 5. Conclusions

**Figure 8:**
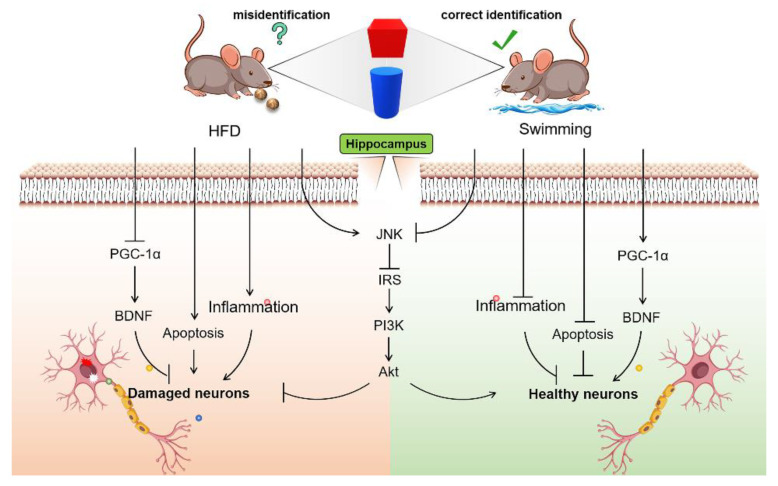
Swimming rescued the impaired cognitive capacity in adolescent mice caused by high-fat diets by suppressing inflammation, alleviating insulin resistance, up-regulating BDNF, and inhibiting apoptosis.

Adolescents require more nutrients and energy, but the consumption of long-term high-fat diets can also lead to body weight gain and impaired learning and memory capacity, which is highly correlated with neuroinflammation, insulin resistance, reduced neurotrophic factors, and increased neuronal apoptosis. Swimming intervention can reverse these abnormal changes to rescue the impaired learning and memory capacity in obese mice by reducing obesity, alleviating hippocampal neuroinflammation, activating insulin signaling, promoting the generation and secretion of neurotrophic factors, and suppressing hippocampal neuronal apoptosis in adolescent mice, with the involvement of the JNK/IRS-1/PI3K/Akt and PGC-1α/BDNF signal pathways (Figure 8).

## Data Availability

The data are available from the corresponding author.
